# Management of myopic traction maculopathy

**Published:** 2025-01-31

**Authors:** Barbara Parolini

**Affiliations:** 1 Head of Vitreoretinal Unit Eyecare Clinic, Brescia, Italy.

**High myopic eyes with myopic traction maculopathy were expected to improve only anatomically after surgery. With early detection and appropriate treatment, however, visual acuity can improve by an average of two lines.**

Myopia, or short sightedness (defined as more than or equal to 0.50 D of myopia) is the leading cause of refractive error, affecting 35% of the population in 2023.^1^

Genes have been identified for myopia^2–5^ that are thought to determine one's susceptibility to environmental factors, including too much time spent on near work,^5^ insufficient time spent outdoors, low levels of vitamin D,^6^ inadequate light exposure, and poor diet. There is evidence emerging that increased time spent outdoors can reduce the risk of developing myopia and – in those with myopia – it can reduce the rate of progression.^5^

High myopia, defined as refractive error above 6 diopters (D) and/or axial length above 26.5 mm (an eyeball longer than normal), affects a growing number of people worldwide, with the highest rates in urban Asian countries (5–9%).^6^

The progressive elongation of the myopic eye leads to three main consequences at the back of the eye: atrophy, neovascularisation, and tractional changes; collectively, these are known as **myopic maculopathy**.

1. **Atrophy** indicates thinning of the choroid. The choroid, stretched by the elongation of the eye, breaks, creating lacquer cracks (see Figure 1 a and b), and then disappears (atrophy, see Figure 1, c and d). This complication is untreatable
2. **Neovascularisation** is the formation of new vessels from the choroid to the retina (Figure 1b and Figure 2). This complication is treatable with intravitreal anti-VEGF injections.
3. **Tractional changes** in the retina, (Figure 1e and f, and Figure 3) are known as myopic traction maculopathy (MTM); this may affect up to 30% of eyes with high myopia.

**Figure 1:**
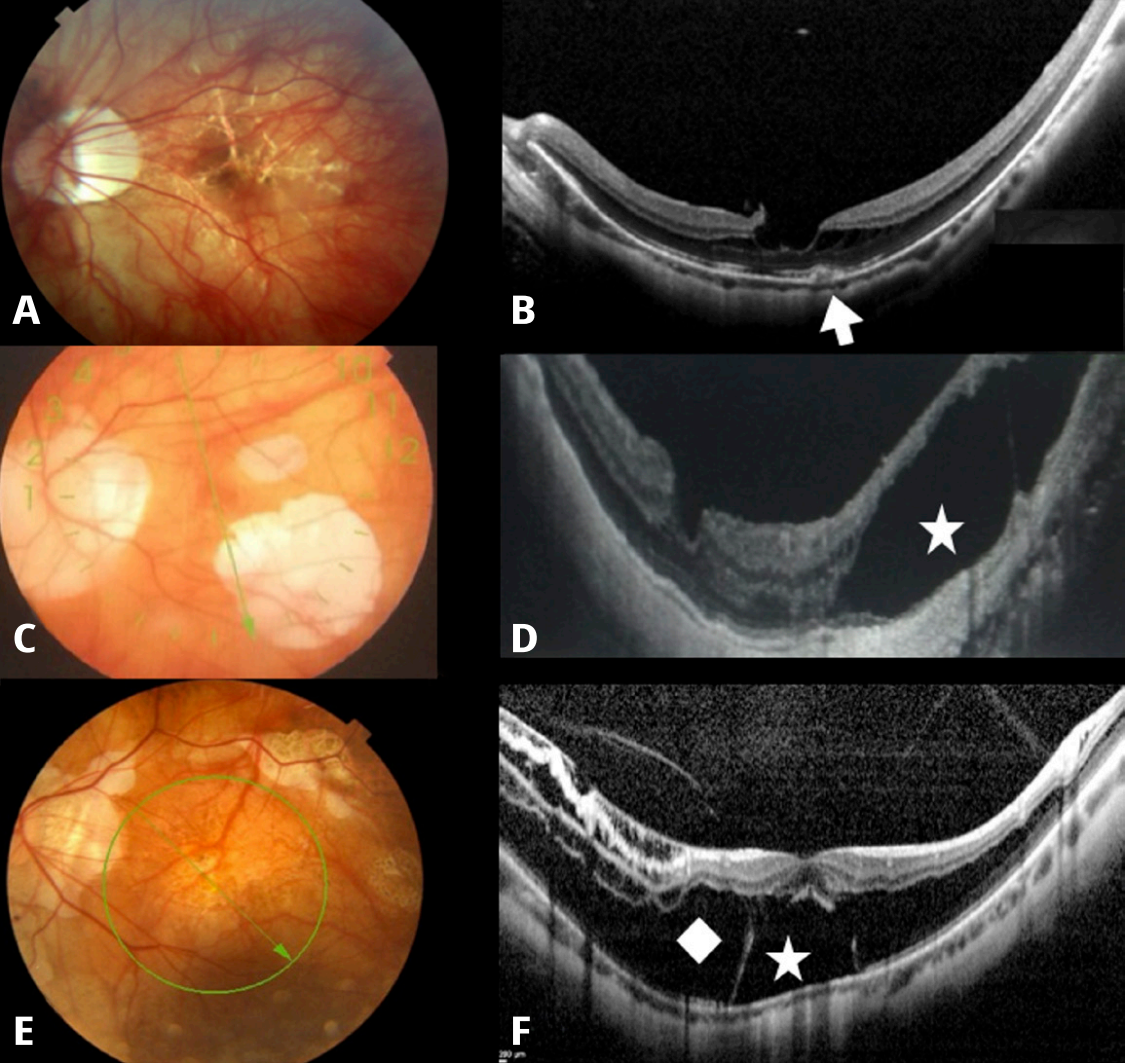
**A:** Colour fundus retinography showing the fundus of an eye with myopic maculopathy and laquer craks (yellowiwsh lines at the posterior pole). **B:** OCT of the same eye in the macula showing the interruption in the choroid with a small choroidal neovascularization (arrow). **C:** Colour fundus retinography showing the fundus of an eye with myopic maculopathy and choroidal atrophy. **D:** OCT of the same eye displayed in C, with high myopia and areas of choroidal atrophy with serous retinal detachment (white star) correspondent to the atrophic area. **E:** Colour fundus retinography showing the fundus of an eye with myopic maculopathy and no apparent lesions. **F:** OCT showing myopic traction maculopathy (MTM) in the form of macular schisis (white diamond) and foveal detachment (white star).

© BARBARA PAROLINI CC BY-NC-SA 4.0

**Figure 2:**
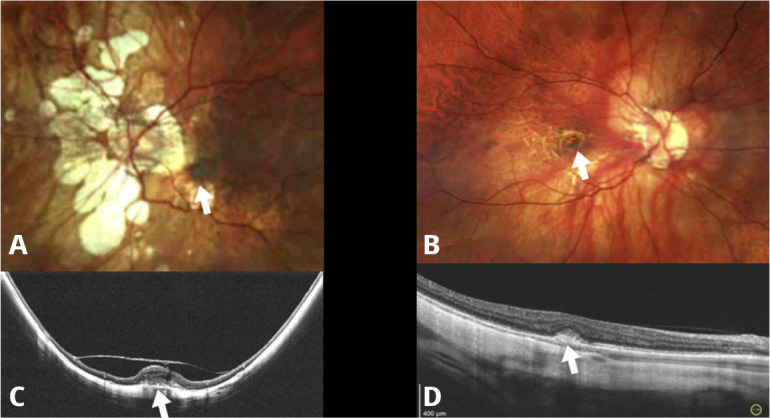
**A:** Color fundus retinography showing the fundus of an eye with myopic maculopathy and whitish atrophy in the peripapillary area. The arrow shows a choroidal neovascular membrane. **B:** Color fundus retinography showing the fundus of an eye with myopic maculopathy. There are no areas of atrophy. The choroidal vessels are visible in the typical tessellated fundus appearance. The arrow shows a choroidal neovascular membrane. **C:** OCT of the same eye displayed in A; the arrow indicates the choroidal neovascular membrane. **D:** OCT of the same eye displayed in A; the arrow indicates the choroidal neovascular membrane.

© BARBARA PAROLINI CC BY-NC-SA 4.0

**Figure 3:**
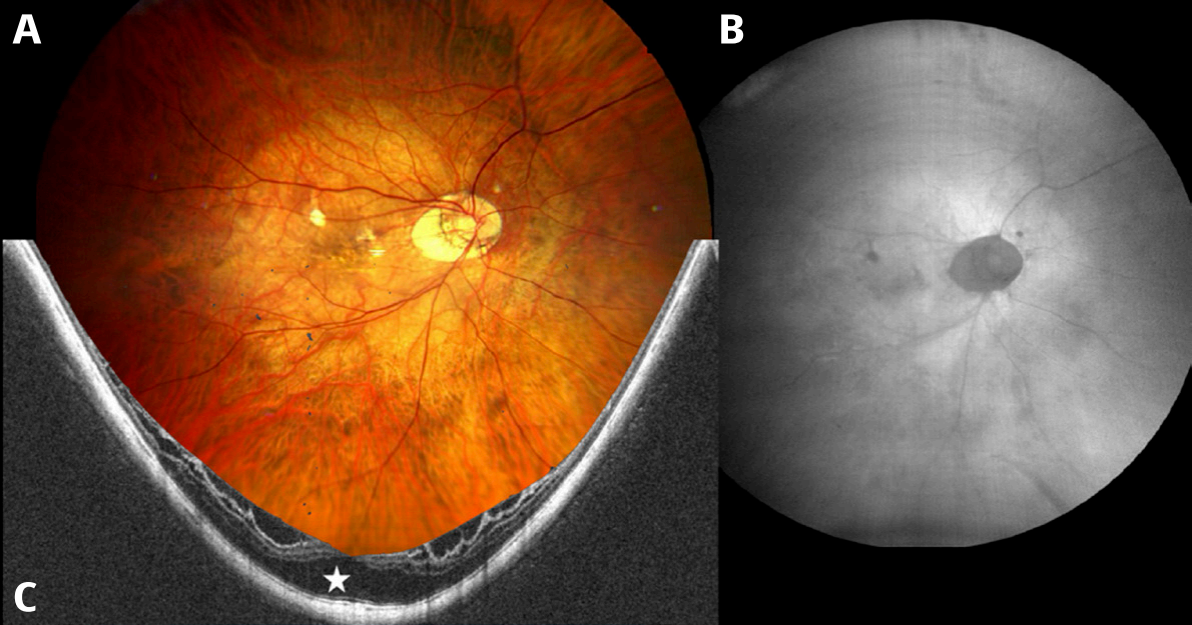
**A:** Colour fundus retinography showing the fundus of an eye with myopic maculopathy and laquer cracks (yellowish lines at the posterior pole) and small areas of whitish atrophy temporal to the papillae and along the superior arcade **B:** Autofluorescence of the same eye. The dark areas corresponds to atrophic zones **C:** Superimposed OCT of the same eye, showing myopic traction maculopathy in the form of macular schisis (white star).

© BARBARA PAROLINI CC BY-NC-SA 4.0

## Myopic traction maculopathy

**Figure 4:**
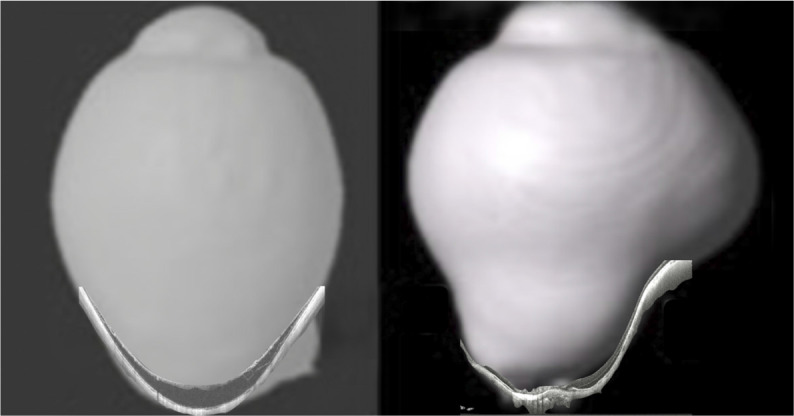
3D magnetic resonance imaging (MRI) of two eyes affected by posterior and lateral staphyloma, elongation of the eyewall and high myopia. The B-scan widefield OCT is superimposed on the posterior pole of the MRI images. The patient on the left has myopic traction maculopathy stage 2a, and the patient on the right has high myopia without traction.

Myopic traction maculopathy (MTM) is the term used to describe the different tractional effects on the macula due to the elongation of the eye as a result of high myopia. MTM may affect up to 30% of eyes with high myopia,^2,3^ especially if a posterior staphyloma is present as an ectasia (or bulging) in the posterior eyewall. The ectasia of the posterior eyewall is visible with widefield OCT or magnetic resonance imaging (MRI), as shown in Figure 4.

© BARBARA PAROLINI CC BY-NC-SA 4.0

MTM is considered a complex disease. But everything is complex only when it is not fully understood.

Until 2020, there was no comprehensive classification in the literature,^4,5,7^ very limited information on pathogenesis and natural history, and no consensus on the complete terminology of the different types of myopic traction maculopathy, nor on treatment.

**Figure 5:**
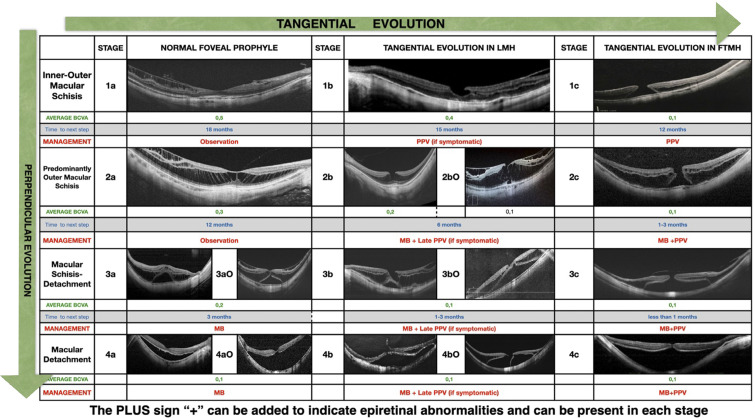
Myopic traction maculopathy staging system.

We analysed hundreds of OCT images of highly myopic eyes, affected by different stages of MTM, over a very long period and then published the MTM staging system (Figure 5). This divides MTM not in types but in stages^8^, to highlight the dynamic and continuously evolving nature of the disease. Next, we offered guidelines on the type and timing of management, customised for each stage.

© BARBARA PAROLINI CC BY-NC-SA 4.0

### The stages of MTM

As the eye elongates and enlarges in progressive myopia, the macula might show signs of anteroposterior traction (traction perpendicular to the foveal plane) and tangential traction (traction at an angle to the foveal plane).

The four rows in the MTM staging system (Figure 5) represent the evolution of the disease in a direction perpendicular to the retina: from inner/outer schisis (splitting of the retinal layers) (stage 1) to predominantly outer schisis (stage 2) to schisis-detachment (stage 3) to complete macular detachment (stage 4). The three columns represent the evolution in a direction tangential to the retina and the fovea: from normal fovea (stage a) to inner lamellar macular hole (stage b) to full-thickness macular hole (stage c).

The outer lamellar macular hole is marked as O and might occur in stage 2, 3, or 4.

The presence of epiretinal abnormalities is marked as + (read as “plus”) and might occur in every stage.

The retina can evolve from stage 1 to stage 4 and from pattern a to c simultaneously or separately. The mean time taken to evolve from one stage to the next is marked in Figure 5, as well as the average visual acuity.

MTM stages might show a spontaneous improvement^4^. However, we determined that, when the eyes are followed for a long time, MTM might start to evolve once again, even after spontaneous resolution.

### How we diagnose myopic traction maculopathy

Indirect ophthalmoscopy and biomicroscopy can identify pathologic myopia but not MTM. OCT is the key instrument to diagnose this disease^1,9^. In fact, the true description of MTM began with the advent of OCT.^1^

When OCT is not available, and the vision is good, we cannot exclude that MTM could be already present. However, usually in early stages, like stages 1a, 1b, 2a, 2b (Figure 5) vision is still in the range of 0,5–1,0 decimal, in the absence of macular atrophy.

When vision starts to decrease in the absence of atrophy, neovascularisation, and haemorrhages, we should suspect MTM in the form of stage 3, 4, or any stage c, even in the presence of a normal fundus appearance, as shown in Figure 1 e and f. These patients should be referred for OCT.

Ideally, all patients with high myopia should be referred for an OCT, if available, no matter what level of vision they have, in order to identify possible myopic maculopathy, classify the stage of myopic maculopathy (if present) and offer appropriate advice for future management.

## How we manage myopic traction maculopathy

Our studies show that, to obtain the best efficacy to safety ratio, patients in the early stages of myopic traction maculopathy, with intact fovea and good vision, should be observed, since progression is slow. For patients with more advanced disease, treatment is required:

- MTM, in the form of schisis and detachment of the macula, can be counteracted by placing a macular buckle which pushes the sclera towards the retina, solving the schisis and detachment.
- Lamellar or full thickness macular hole can be counteracted by pars plana vitrectomy (PPV) which creates a force pointing toward the centre of the fovea.

In summary, when vision is good, the patient should just be observed but with periodic OCT. In the presence of a macular detachment, a macular buckle should be applied.

If a macular hole is present, consider vitrectomy.

One great advantage of using a macular buckle to solve the schisis and detachment secondary to MTM is that it avoids the use of silicone oil, often proposed when treating recurrent retinal detachments in MTM. Standard or heavy silicone oil in highly myopic eyes inevitably leads to secondary glaucoma.

### Macular buckle

The macular buckle is a device that shortens the eye. The surgical technique aims to counteract the pulling effect exerted on the retina by the elongation of the sclera. The buckling side of the device is placed behind the posterior pole, in order to push the sclera anteriorly.

Different models of macular buckle have been proposed.^10^ Surgery may be performed under general or local anaesthesia. For local anaesthesia, we prefer sub-Tenon's anaesthesia with a blunt cannula to avoid the potential risk of scleral perforation with a retrobulbar injection in highly myopic eyes. The surgical technique is shown in this video: https://youtu.be/WdIjLCHbYtE

When following these guidelines, the prognosis of surgery is good, and the best corrected visual acuity improves with an average of 2 lines. It is particularly important to highlight this achievement, because, currently, high myopic eyes with myopic traction maculopathy are expected to improve only anatomically, not functionally, after surgery.^11^

**Case study**

**Figure 6:**
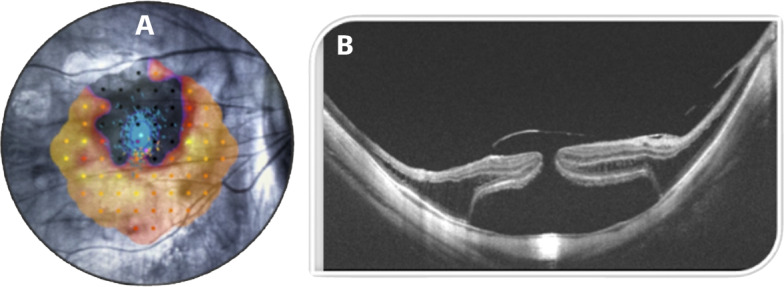
Patient with MTM stage 3c, best corrected visual acuity 6/60. Preoperative image. **A:** Microperimetry showing severe scotoma and low macular sensitivity. **B:** OCT showing the macular schisis and detachment associated with a full-thickness macular hole.

A 56-year-old woman was affected by MTM stage 3c with schisis and detachment of the macula associated with a full thickness macular hole. The preoperative microperimetry showed a black central scotoma corresponding to the area of detachment and hole (Figure 6).

© BARBARA PAROLINI CC BY-NC-SA 4.0

**Figure 7:**
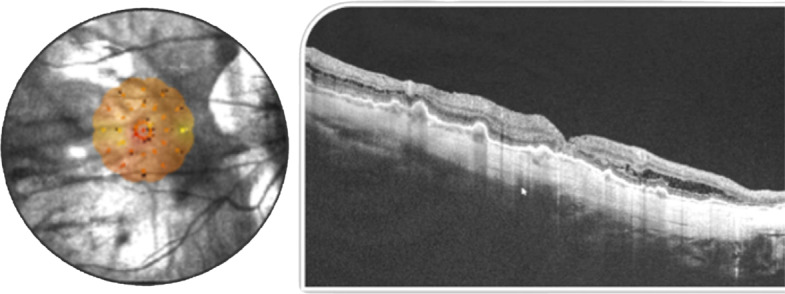
The same patient 5 years after surgery with macular buckle and vitrectomy, with great improvement in best-corrected visual acuity to 6/9 and no scotoma in microperimetry (left).

Figure 7 shows the long-lasting improvement in vision and microperimetry 5 years after surgery with macular buckle to treat the detachment, with vitrectomy and an internal limiting membrane flap^12^ to treat the macular hole, with disappearance of the scotoma. The image on the right shows the postoperative OCT with flat retina and closed hole.

© BARBARA PAROLINI CC BY-NC-SA 4.0
